# Natural occurrence and distribution of entomopathogenic nematodes (Steinernematidae, Heterorhabditidae) in Viti Levu, Fiji Islands

**DOI:** 10.21307/jofnem-2020-017

**Published:** 2020-03-19

**Authors:** Sumeet Kour, Uma Khurma, Gilianne Brodie, Selcuk Hazir

**Affiliations:** 1 School of Biological and Chemical Sciences, Faculty of Science, Technology & Environment, The University of the South Pacific, Fiji Islands; 2 Institute of Applied Sciences, The University of the South Pacific, Fiji Islands; 3 Department of Biology, Faculty of Arts and Sciences, Adnan Menderes University, Aydin, Turkey

**Keywords:** Entomopathogenic nematodes, Biological control, *Heterorhabditis*, Distribution, Survey, Fiji Islands

## Abstract

In Fiji, little or no attention has been given to entomopathogenic nematodes (EPNs) in biocontrol programs due to the lack of awareness about their occurrence and distribution in Fiji. A survey of EPNs was conducted for the first time in Fiji Islands in 2012 and 2013, throughout the eight provinces in Viti Levu to determine the occurrence and distribution across different habitat in Viti Levu. The soil samples from various habitats were collected and assayed for the presence of EPN using *Galleria mellonella* as baits. EPNs were recovered from five out of seven provinces with 35 positive sites (7.3%) out of 478 sites sampled. The only EPN genera encountered was *Heterorhabditis. Steinernema* was not isolated from any of the samples. Characterization of isolates was done by using morphometric and molecular examinations and isolates were identified as *Heterorhabditis indica. H. indica* isolates were primarily recovered from leeward side of the Viti Levu Island along the coastline and riversides, being more prevalent in lighter soil with pH > 6. Further, this study found significant association between habitat type, soil type, soil pH, average annual rainfall and EPN occurrence. This is the first record of naturally occurring EPNs in Fiji. The found nematodes will serve as the basis for efficacy screening with the ultimate aim of delivering effective, more sustainable and environmentally safe control for agricultural pests in Fiji.

Entomopathogenic nematodes (EPNs) species belonging to the genera *Steinernema* Travassos, 1927 and *Heterorhabditis* Poinar, 1975 and their symbiotic bacteria from genera *Xenorhabdus* and *Photorhabdus*, respectively, are lethal parasites of soil inhibiting insects (Shapiro-Ilan et al., 2017). Globally, EPNs are being widely researched as promising biocontrol agents for wide range of agricultural pests (Lacey et al., 2015). Because of the increasing awareness of EPN as an effective non-chemical alternative to control insect pests, many surveys have been and are being carried out across the globe to isolate new species and/or population of these nematodes that are either more virulent and/or locally adapted to the region’s environmental conditions (Yan et al., 2016; Tarasco et al., 2015). Interest has also been shown in isolating novel species and strains that have characteristic traits, for example species with: improved heat tolerance (Shapiro et al., 1996; Wetchayunt et al., 2009), desiccation tolerance (Salame et al., 2010; Solomon et al., 1999), high reproductive potential (Salame et al., 2010), foraging ability (Noosidum et al., 2010), virulence (Shapiro-Ilan et al., 2009; Yu et al., 2010) and cold tolerance (Ivanova et al., 2001; Wright and Jackson, 1988) have been found in recent surveys. Discovery of new isolates/strains and species has substantially bolstered the commercial success of EPN biocontrol agents against pests (Lacey and Georgis, 2012; Shapiro-Ilan *et al.*, 2002).

In Fiji, there are several coleopteran, dipteran, lepidopteran and isopteran species that are serious agricultural pests and cause great economic losses (Furlong, 2010; Moxon et al., 2008; Waterhouse, 1997). Fiji has a long history of biocontrol of insect pests which began in early 1910s (Kamath, 1979). However, by 1960s, farmers started using pesticides to control insect pests (Autar, 2000) which has resulted in emergence of issues like development of insect resistance to chemical pesticides (Atumurirava and Furlong, 2011) and rise in health problems in the farmers that rely on chemical pesticides for crop protection (Szmedra, 2002) forcing the policy makers to shift their focus from chemical intensive pest management to alternative control strategies or integrated pest control methods. Since the 1980s, focus has been shifted to more integrated approach with greater emphasis to the use of biocontrol agents together with the use of safer pesticides (Autar, 2000). Various biocontrol agents are being increasingly explored by Fiji, e.g. parasitoids (Bale et al., 2008), virus (Jackson, 2009), predators (Upadhyay et al., 2012) and fungus (Day et al., 2013; Pene et al., 2007) and have been successfully employed to control insect pests and weeds. However, in Fiji, little or no attention has been given to EPNs in biocontrol programs due to the lack of awareness about EPNs and of local expertise in this field. There are several target pests in Fiji that can be potentially managed with EPNs and this area of research remains largely unexplored. Therefore, the exploration of indigenous EPN species was carried out for the first time to acquire a new resource for biological control of insect pests.

## Material and methods

### Site description

**Figure 1:**
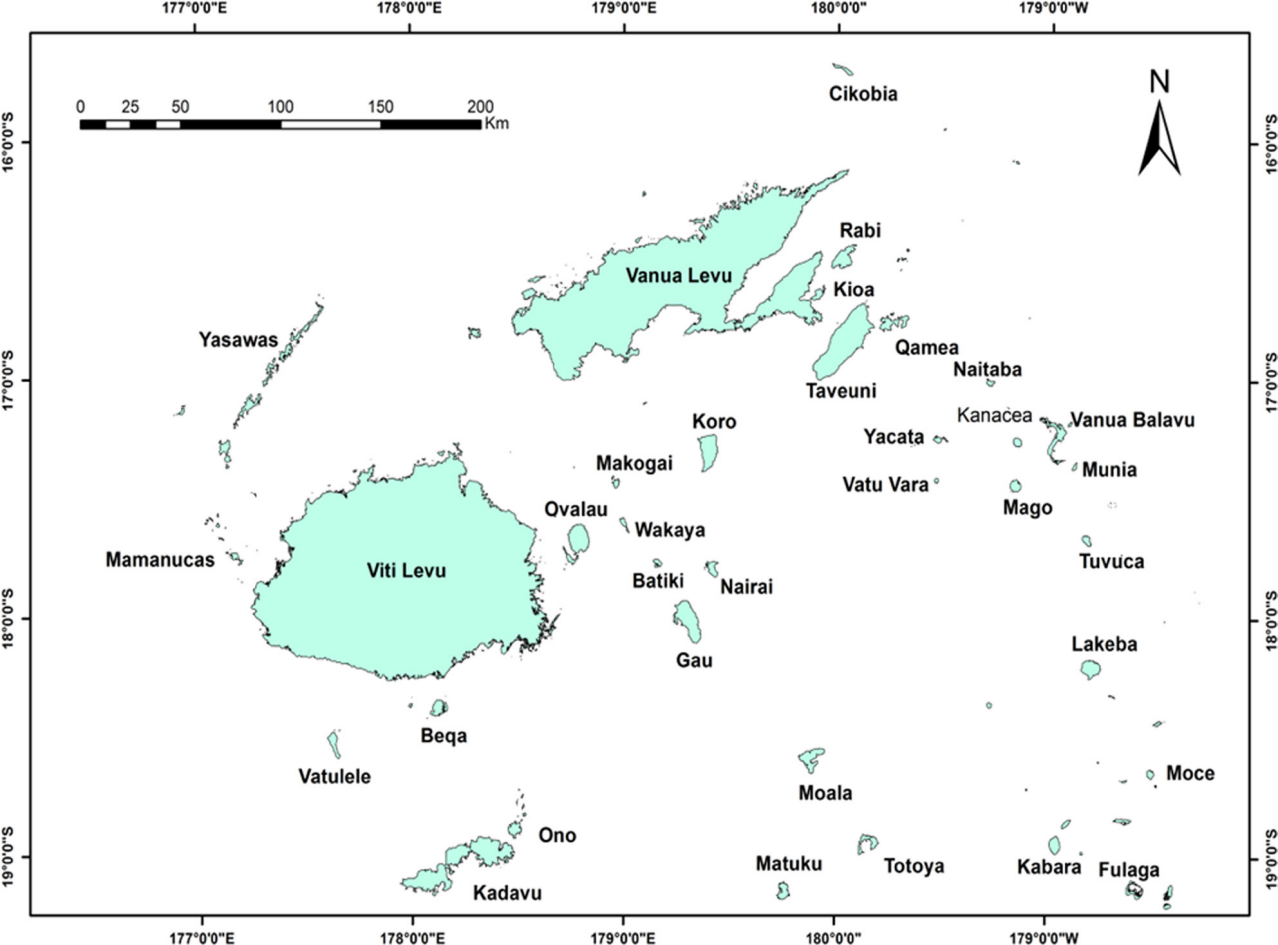
Map of Fiji Islands.

The Fiji Islands (Fig. 1) lie in the South Pacific Ocean between 174°E and 178°W longitude and 12° and 22°S latitude (Morrison et al., 1990). The Fiji archipelago consists of 330 islands covering a land area of 18,376 km^2^ (Mataki et al., 2006). Out of 330 islands only 100 are inhabited. Most of the islands belonging to this archipelago are of volcanic origin. Viti Levu (10.642 km^2^) is the oldest and the largest island of this Archipelago, and makes 57% of the land area. Around 65% of the Viti Levu is characterized by steep mountainous terrain with slope of 18° to 70°, whereas 20% is covered by gentle slope and 15% by flat land which is restricted only to the lowland valleys (Ash and Ash, 1984; Morrison et al., 1990). In Viti Levu, plains and plateau have elevation ranging between 100 and 500 m above sea level (a.s.l.) (Morrison et al., 1990), whereas the mountain height ranges between 900 and 1,320 m a.s.l. These mountains divide the island of Viti Levu into a windward south-eastern side and a leeward western side (Manueli et al., 1999; Neall and Trewick. 2008). Due to the orographic effect of these mountains, moisture laden southeast trade winds cause average annual rainfall between 305 and 345 cm on the “wet” windward side, whereas the western leeward side remains dry with average annual rainfall between 165 and 229 cm. Wet windward side of the island is covered with moist tropical forest, whereas the dry leeward side is dominated by grass, fern and introduced invasive elements (Evenhuis and Bickel, 2005). Fiji’s climate is humid tropical and experiences two seasons: winter (between May and October) and summer (between November and April). The winters are cool and dry with average temperature range of 19°C to 22°C while summers are hot and humid with average temperature range of 31°C to 34°C.

### Sampling method

The survey was conducted in Viti Levu. Soil samples were randomly collected from diverse habitats from March 2012 until July 2013 covering all the eight provinces (Rewa, Ba, Tailevu, Ra, Nadroga-Navosa, Namosi, Serua and Naitasiri) of Viti Levu. In total, 478 sites were sampled. In each province, the samples were collected from coastline, lowland (cultivated and natural habitats), and highland areas representing different habitat types. Viti Levu has a very steep mountainous terrain and the locations for sampling were also chosen based on the road accessibility and reachability. The approximate distance between two sampling sites was 3 to 4 km. At each sampling site (≈100 m^2^), 12 to 15 soil samples were taken from surface up to the depth of 20 to 25 cm using a soil corer with a bore size 3.2 cm. The samples were pooled together and thoroughly mixed to make a final composite site sample of approximately 2 kg. GARMIN eTrex Vista GPS unit (global positioning system) was used to record the sampling site positions. Each composite soil sample was placed in a zip lock plastic bag and labeled for site name and date of collection. Notes on the surrounding vegetation at the sampling site were also taken. The environmental habitat immediately surrounding each sampling site was recorded as one of the following categories: Grassland, Garden/Park, Farmland, Forest, Riverside, Coastline/Dunes and Roadside verge. To our knowledge, no EPN based products were ever released at any of the 478 sites sampled. Site locations were plotted on base map using software ArcMap version 10.4.1. The soil sampler was sterilized with 70% ethanol before leaving the sampling site.

### Isolation of entomopathogenic nematodes

The soil samples were processed on the site to isolate native EPNs using wax moth *Galleria mellonella* (Lepidoptera: Pyralidae) larvae as the insect bait (Bedding and Akhurst, 1975). Freshly harvested *G. mellonella* larvae (last instar) which were immersed in hot water (60°C) for 20 sec to prevent them from forming a cocoon were used as bait. Immediately after collecting, each soil sample was placed in a 250 ml plastic container with a lid, in which six *G. mellonella* larvae were added in the field. Three larvae were placed at the bottom and three in the middle of the soil. This was replicated three times. Therefore, in total for each site 750 ml of soil was baited with 18 larvae. When needed, the soil samples were moistened with distilled water to facilitate the movement of IJs (Nielsen et al., 2004). The plastic containers (with soil and bait) and leftover soil were placed in an insulated container and transported to laboratory. In laboratory, the plastic containers were placed upside down and incubated at room temperature (≈25°C). After 48 hr of incubation, the containers were checked daily for two weeks and any dead larvae were removed from the soil, and the color of the cadaver was recorded. Dead larvae were thoroughly rinsed with distilled water and transferred to modified White Traps (White, 1927). White traps were kept at room temperature (≈25°C) until the emergence of the IJs and were tested for Koch’s postulates. The emerging IJs were collected in sterile deionized water and stored in a 750 ml tissue culture flask at 15°C for morphological and molecular characterization. The EPN laboratory populations were maintained by passing through the host *G. mellonella* larvae every 3 to 4 months’ interval.

### Identification of nematodes

To identify isolates of EPN at species level both morphometric and molecular examinations were made. Molecular analysis was considered as the primary approach. Molecular characterization of the isolates was performed by analysis of the ITS rDNA sequences.

#### Molecular characterization

Individual adult nematodes were dissected from infected *G. mellonella* cadavers. Total genomic DNA was extracted from a single hermaphroditic female as described by Kaspi et al. (2010). DNA samples were stored at −20°C until the next stage of processing. Polymerase chain reaction (PCR) was used to amplify the region of nuclear rDNA. The targeted segment which included: partial 18 S 3´ end, ITS1, ITS2, 5.8 S subunit, and partial 28 S 5´ end were amplified using primers no. 93, 5´ TTGAACCGGGTAAAAGTCG (forward primer) and no. 94, 5´ TTAGTTTCTTTTCCTCCGCT (reverse primer) designed by Nadler et al. (2000). The PCR-product was purified using ExoSAP-IT kit (Affymetric, Inc.). The purified products were sent to a sequencing service in Seoul, Korea (Macrogen, Korea). The sequencing raw data were visualized, edited and assembled by using DNABaser Sequence Assembler software version 3.5.4.2 (http://www.dnabaser.com/index.html). The full length sequence of native isolates was confirmed to comprise of ITS1, 5.8 S and ITS2 region by alignment with the corresponding sequence of *C. elegans* (Ellis et al., 1986). The consensus sequences obtained were deposited in GenBank. For species identification, the obtained sequences were used for a Basic Local Alignment Search Tool (BLAST) search at National Center for Biotechnology Information website http://blast.ncbi.nlm.nih.gov/Blast.cgi and were compared with the sequences of the species already deposited in international GenBank database. Multiple alignments of sequences obtained with GenBank ITS sequences from Heterorhabditis species gene sequences were obtained using Clustal X (version 1.83) (Larkin et al., 2007). Maximum parsimony analysis was conducted in MEGA 6.06 (Tamura et al., 2013). Bootstrap analysis was carried out with 1,000 data sets. The phylogenetic analyses provided further support for species identification. The genetic variability among Fiji populations was also analyzed using ITS1 and ITS2 regions.

#### Morphometric characterization (light microscopy)

Based on the result of molecular analyses, selected isolates RATA, SSSX and DUNF were subjected to morphometric examination. Nematodes were reared in last instar of *G. mellonella* larvae as described by Nguyen (2007). For each isolates to be examined, 20 specimens from each stage were randomly collected from different *G. mellonella* cadavers. First generation hermaphrodites, and second generation males and females were heat killed at 60°C in normal saline and fixed in triethanolamine formalin (TAF) as described by Hominick et al. (1997). Once fixed, nematodes were then processed to anhydrous glycerin as per Seinhorst (1959). The nematodes were mounted in desiccated glycerin on glass slides. IJs were examined live by placing on glass slide in a drop of water and covered with a coverslip. The slides were examined after a few minutes when the IJs became motionless. The live IJs were directly used for microscopic observations as key characteristics such as “excretory pore” and “base of esophagus” were clearly visible as opposed to in heat killed and TAF fixed IJs. All measurements were made using compound light microscope Olympus BX51 equipped with digital image software. The following morphometric characters were analyzed: total body length, greatest body width, distance from anterior end to excretory pore, distance from anterior end to nerve ring, distance from anterior end to base of esophagus, tail length, anal body width, spicule length, gubernaculum length, testis reflexion, values of ratio a, ratio b, ratio c, V%, D%, E%, SW% and GS%.

### Soil characterization

Soil parameters like temperature and pH were recorded *in situ* at the depth of 10 to 12 cm during soil sample collection by using Hanna HI 99121 pH meter. Soil volumetric moisture (%) was recorded *in situ* by using soil moisture meter LT Tutron PMS-714 EZTECH PMS-714. Facilities at Fiji Agricultural Chemistry Laboratory, Koronivia Research Station, Nausori were used for measurement of soil particle size and measurement of soil organic content. Soil particle size analysis was performed to calculate the % of sand, silt and clay in soil sample and to determine the overall soil texture at each sample site. The method adapted from the New Zealand Standard NZS4402 (Standards Association of New Zealand, 1980) along with the method described by Claydon (1989) was used to calculate Sand/ silt/clay percentages each sample. The New Zealand classification system was followed to classify soil particle as: clay (<0.002 mm), silt (0.002-0.06 mm) and sand (0.06-2.0 mm) (Hewitt, 1992). The New Zealand soil triangle and soil texture class (clay, clay loam, fine sand, loam, loamy fine sand, sandy clay, sandy clay loam, sandy loam, silt loam, silt clay and silty clay loam) as defined by Milne (1995) was used to determine the texture of the sampled site. Total organic matter for each soil sample was determined by wet oxidation as described by Walkley and Black (1934).

### Meteorological data

The long-term mean annual rainfall, long-term mean annual maximum air temperature and minimum air temperature data of the different localities from 1971 to 2000 were obtained from 11 weather stations closest to the sampling sites with the help of Nadi center of Fiji Meteorological service. The data from 11 weather stations (Laucala Bay, Tamavua, Nausori, Koronivia, Nadi Airport, Rarawai, Penang, Nacocolevu, Lautoka, Navua and Monasavu) in Viti Levu were used in current study.

### Data analysis

The percentage occurrence of EPNs is reported in terms of recovery frequency (number of positive samples/number of total samples). The occurrence of EPNs was then plotted against habitat type, soil characters, geographical location and environmental variables. Relationships between the occurrence of EPNs and categorical variables (i.e. climatic zone, habitat type, province, and soil texture) were statistically determined using Crosstabs and Pearson’s χ2 test of Independence at α = 5% level of significance (Agresti, 1996). For continuous variables like soil parameters (soil moisture, soil pH, soil organic content and soil temperature) and environmental variables (average annual rainfall, average annual temperature max. and min.), a test for normality was performed to see if test assumptions are not violated. All the continuous variables violated the normality assumptions and hence, non-parametric Mann–Whitney Test was used to study any relationships between the occurrence of EPN and the continuous variables. Statistical analysis was performed using SPSS 21.0 software for Windows XP.

## Results

### Occurrence of entomopathogenic nematodes by province

**Figure 2:**
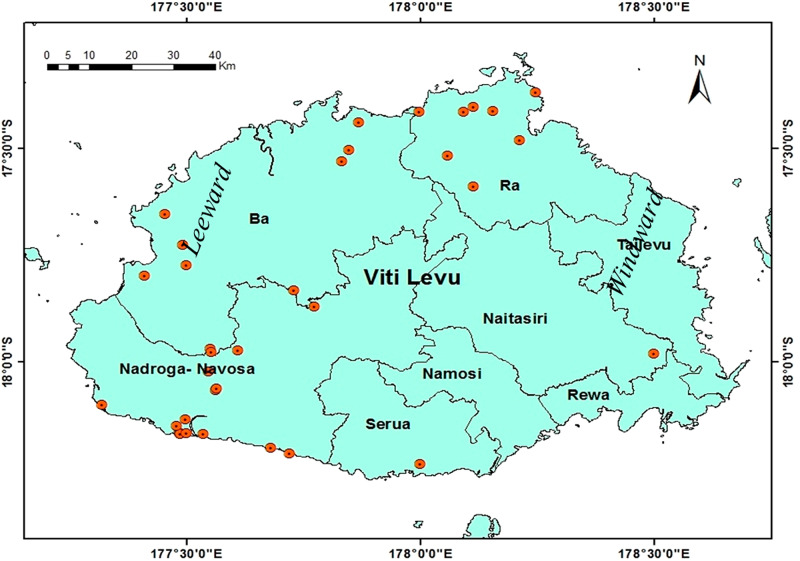
Map of Viti Levu showing sites where entomopathogenic nematodes were found.

EPNs were isolated from 35 of 478 (7.3%) sites sampled around Viti Levu (Fig. 2). All the 35 sites were positive for the presence of the *Heterorhabditis* species. None of the sites was positive for *Steinernema.* The highest number of positive samples was recorded from the Nadroga-Navosa province (18.2%), followed by Ra (12.3%) and Ba (4.5%). Soil samples collected from Namosi, Rewa and Tailevu were negative for EPN (Table 3).

### Molecular characterization

**Figure 3:**
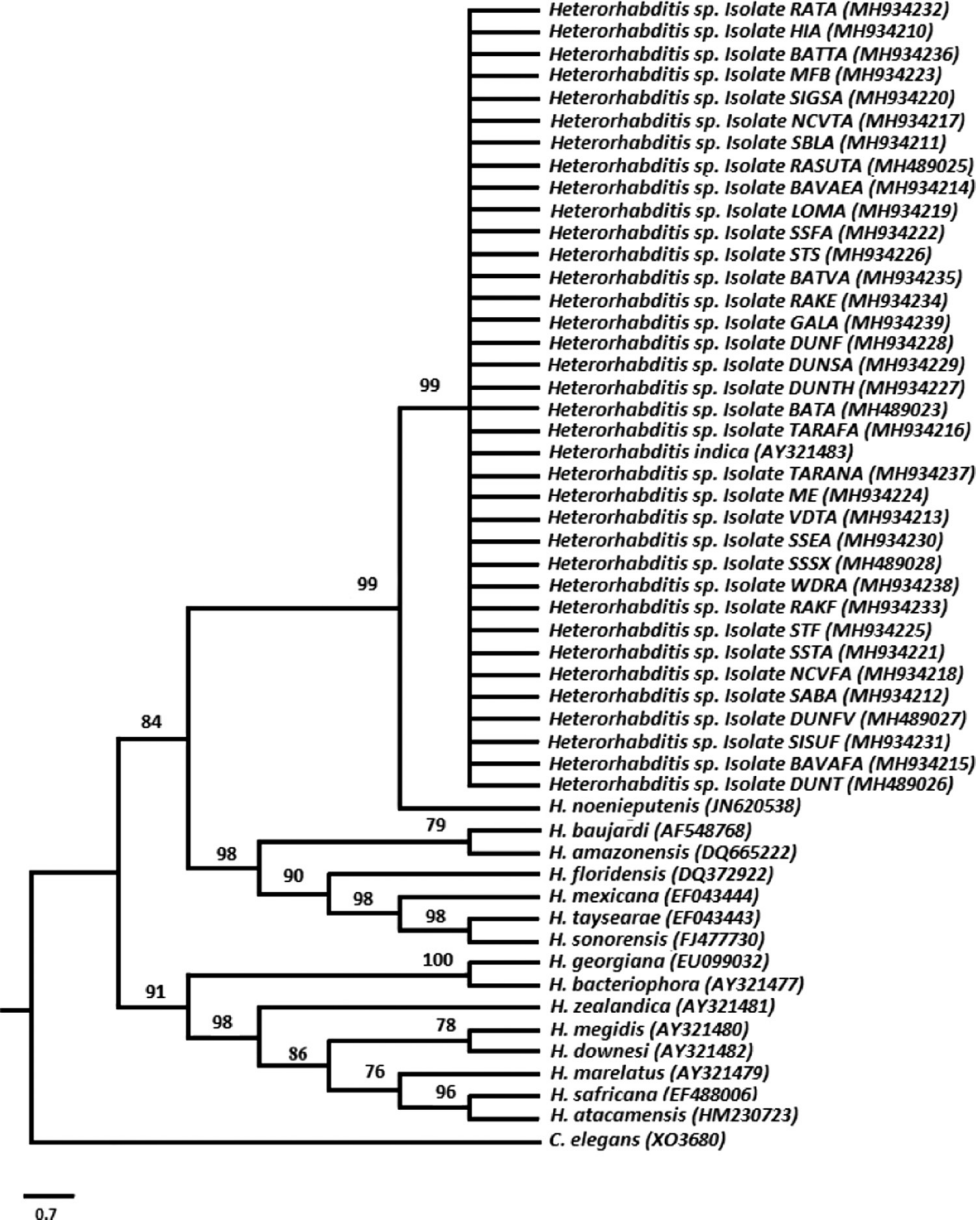
Maximum parsimony tree inferred from sequence of ITS rDNA regions in 17 known *Heterorhabditis* species and native isolates of *Heterorhabditis* spp., with *C. elegans* as out group. Numbers at the nodes represent bootstrap values.

PCR of the entire ITS region amplified a single band composed of the partial 18 S, ITS1, 5.8 S, ITS2 and partial 28 S. The sequence length of ITS region of native isolates from Viti Levu varied from 771 to 989 base pairs. A BLAST search of GenBank database using ITS region of native isolates indicated that all isolates belong to the genus *Heterorhabditis* and showing 100 to 99% similarity with the *Heterorhabditis indica* species isolated from different geographical regions of the world. The next closest species is *H*. *neonieputensis* (#JN620538), with 98% sequences identity. Phylogenetic analysis grouped native isolates with *H. indica* and confirms the identity of native isolates (Fig. 3). Comparison between sequence of native isolates and *H. indica* (AY321483) shows that native isolates differ from each other and *H. indica* (AY321483) by eight base pair difference; one transition (A-G) at position 45 and one transversion at position 329 in ITS1 region; two transversion (T-A) at position 586 and 587, three transition (G-A) at position 589, 629 and 718 and a gap at 629 in ITS2 region. The difference in the ITS region for native isolate and *H. indica* (AY321483) has been highlighted in Table 1.

**Table 1.**
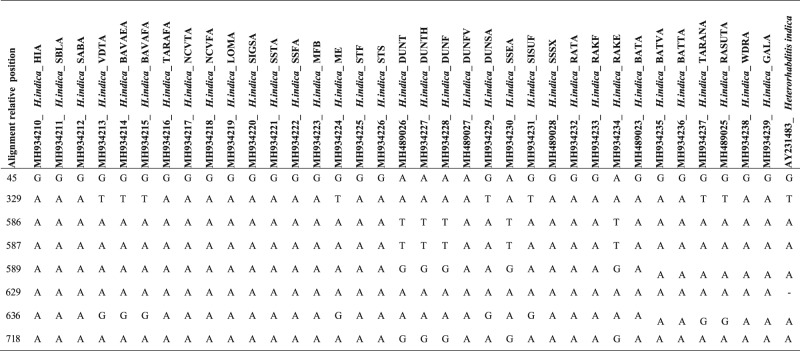
Nucleotide difference between population of *Heterorhabditis* spp. from Fiji.

### Morphometric characterization

Body length of IJ is from 520 to 630 μm with long tail (77.5-130 μm). Except for isolate DUNF, value for EP (98.1-99.6 μm) is like type species *H. indica*. Ratio c is also comparable to the range of *H. indica*. Male body measured from 748 to 1,137 μm which is longer than the length given for type species *H. indica*. Testis reflexion measured from 91 to 92, like type species *H. indica*. Similarly, the both female and hermaphrodite of native isolates were much bigger in size for type species *H. indica*. The comparison of the measurements of the native isolates with that of the type population of *H. indica* are given in Table 2.

**Table 2.**
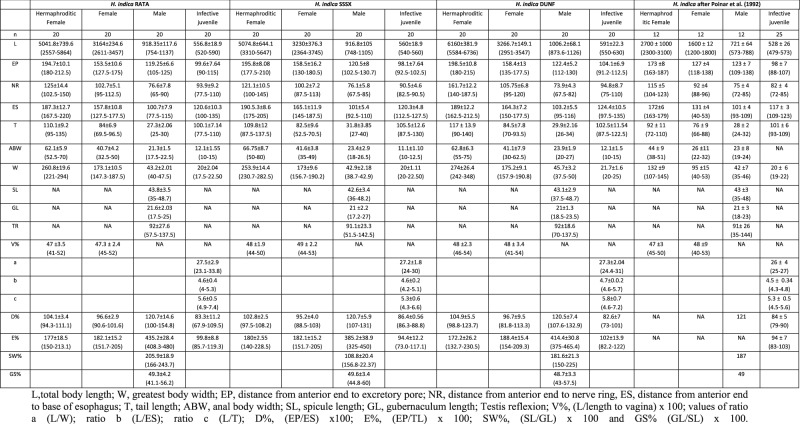
Comparative table of morphometric of hermaphroditic females, females, males and infective juvenile of the native isolates RATA, SSSX and DUNF from Viti Levu and hermaphroditic females, females, males and infective juvenile of *H. indica* (in μm, mean ± SD, and range in parenthesis).

### *H. indica* occurrence and habitat type

Coastline and sand dunes were most *H. indica* rich habitats (25%), followed by river banks (15.1%). Gardens and parks were negative for *H. indica* (Table 3). In Viti Levu, habitat type had significant association with *H. indica* presence, (*χ* ^2^ (6, *N* = 478) = 36.01, *p* < 0.001).

**Table 3.** Occurrence of entomopathogenic nematodes in different province and habitat types.

Categories	Total samples	Positive samples	Recovery frequency (%)
*Province*			
Ba	154	7	4.5
Nadroga-Navosa	99	18	18.2
Naitisiri	43	1	2.3
Namosi	19	0	0
Ra	65	8	12.3
Rewa	33	0	0
Serua	27	1	3.7
Tailevu	38	0	0
*Habitat type*			
Coastline/Dunes	40	10	25
Farm	71	2	2.81
Forest	125	2	1.6
Gardens/Park	10	0	0
Grassland	136	7	5.14
Riverside	79	12	15.1
Roadside verge	17	2	11.7

### *H. indica* occurrence and soil characteristics

Occurrence of *H. indica* was examined with regards to soil pH, moisture, soil temperature, soil texture and organic matter content. Soil texture analysis showed that positive sites have relatively high sand content. *H. indica* was found in fine sand (58.3%), loamy fine sand (24%) and sandy loam (23.2%), and were absent in clay, silty clay, silty loam, silty clay loam and clay loam soil (Table 4). The occurrence of *H. indica* showed highly significant association with soil type (*χ* ^2^ (10, *N* = 478) = 103.2, *p* < 0.001). The soil temperature of positive sites ranged from 24 to 30.9°C (Table 4). The largest percentage of *H. indica* isolates were found in soils with temperature ranging from 30 to 31°C (17.7%). The pH of soil samples harboring *H. indica* ranged from 5.0 (acidic) to 7.5 (slightly alkaline). The organic content of the positive samples varied from 1 to 11.2%. The largest percentages of *H. indica* isolates were found in soils with pH ranging from 6.0 to 6.5 (12.2%) and organic content more than 9% (50%) (Table 4). The soil moisture level for positive soil ranged from 6 to 24% with largest percentage of positive samples found in soil with moisture level >21% (18.1%) followed by 18 to 21% (8.3%) (Table 4). Except for soil pH (*U* = 6141, *p* = 0.04), other factors like moisture level (*U* = 7395.5, *p* = 0.65) and organic content (*U* = 7100.5, *p* = 0.40) showed no significant association with *H. indica* occurrence.

**Table 4.** Occurrence of *H. indica* in Viti Levu with different soil physical & chemical parameters.

Categories	Total samples	Positive samples	Recovery frequency (%)
*Sand content (%)*			
<30	142	0	0
30-45	132	1	0.75
45-60	87	5	5.7
60-75	67	15	22.3
75-90	41	8	19.5
>90	9	6	66.6
*Silt content (%)*			
<20	177	26	14.6
20-30	150	7	4.6
30-40	92	0	0
40-50	43	2	4.6
50-60	12	0	0
>60	4	0	0
*Clay content (%)*			
<20	126	31	24.6
20-30	114	4	3.5
30-40	137	0	0
40-50	60	0	0
50-60	29	0	0
60-70	11	0	0
>70	1	0	0
*Soil texture*			
Clay	96	0	0
Clay loam	124	0	0
Fine sand	12	7	58.3
Loam	27	1	3.7
Loamy fine sand	25	6	24
Sandy clay	9	0	0
Sandy clay loam	83	5	6
Sandy loam	69	16	23.2
Silt loam	10	0	0
Silty clay	12	0	0
Silty clay loam	11	0	0
*Soil pH*			
4.0-4.5	7	0	0
4.5-5.0	22	0	0
5.0-5.5	58	1	1.74
5.5-6.0	104	5	4.8
6.0-6.5	153	19	12.4
6.5-7.0	89	9	10.11
7.0-7.5	37	1	2.7
>7.5	8	0	0
*Soil organic content*			
<3%	236	22	9.3
3-5%	156	7	4.4
5-7%	73	2	2.7
7-9%	9	2	22.2
>9%	4	2	50
*Soil moisture*			
3-6%	1	0	0
6-9%	11	0	0
9-12%	189	15	7.9
12-15%	154	10	6.4
15-18%	76	5	6.5
18-21%	36	3	8.3
>21%	11	2	18.1
*Soil temperature*			
<26°C	68	3	4.41
26-27°C	78	4	5.12
27-28°C	78	5	6.41
28-29°C	80	7	8.7
29-30°C	66	5	7.5
30-31°C	62	11	17.7
>31°C	46	0	0

### *H. indica* occurrence and climatic factors

The majority of the positive samples were from leeward side of Viti Levu (Fig. 2). The occurrence of *H. indica* showed highly significant association with climatic zone (*χ* ^2^ (1, *N* = 478) = 6.65, *p* < 0.05). The greatest percentages of soil samples positive for *H. indica* were recorded in areas with average annual rainfall between 1,700 and 2,300 mm (24%) and between 2,300 and 2,900 mm (9%) (Table 5). Similarly, the greatest percentages of soil samples positive for *H. indica* were recorded in areas with average maximum annual air temperature between 29.0 and 29.5°C (27%) followed by 29.5 to 30.0°C (8.1%) (Table 5). No sites with average annual minimum temperature less than 20°C were positive. The greatest percentages of positive soil samples recorded had an average minimum annual air temperature between 20 and 21°C (13.3%) followed by 23 to 24°C (4%) (Table 5). There was a significant association between *H. indica* occurrence and average annual rainfall (*U* = 4144, *p* < 0.001), whereas average minimum annual air temperature (*U* = 7575, *p* = 0.82) and average maximum annual air temperature (*U* = 6387, *p* = 0.079) had no significant association with *H. indica* occurrence.

**Table 5.** Distribution of *H. indica* in Viti Levu at different environmental variables.

Categories	Total samples	Positive samples	Recovery frequency (%)^a^
*Climatic zone*			
Leeward	255	26	10.1
Windward	223	9	4.0
*Mean annual rainfall (mm)*			
<2,300	218	24	11
2,300-2,900	99	9	9
2,900-3,500	91	1	1
3,500-4,100	52	1	1.9
>4,100	18	0	0
*Av annual max air temp (°C)*			
<28.0	18	0	0
28.0-28.5	70	1	1.4
28.5-29.0	73	1	1.3
29.0-29.5	175	27	15.4
29.5-30.0	49	4	8.1
30.0-30.5	72	2	2.7
>30.5	21	0	0
*Av annual min air temp (°C)*			
<19	18	0	0
19-20	73	1	1.3
20-21	148	20	13.5
21-22	80	1	1.25
>22	159	13	8.1

A complete list of the EPN isolate found, with the indication of the locality, habitat, associated vegetation and soil texture for each of them, the accession number in GenBank is summarized in Table 6.

**Table 6.**
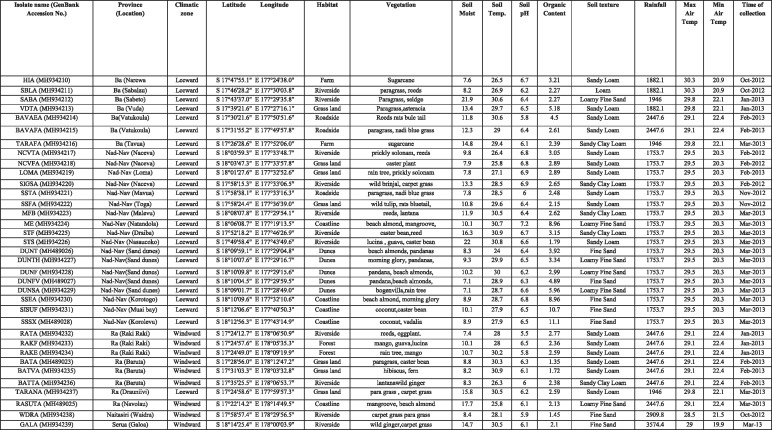
Occurrence of *H. indica* in Viti Levu.

## Discussion

Through this survey the exploration of native EPN species was carried out for the first time in Fiji Islands and was restricted to the main island Viti Levu. The recovery frequency of 7.3% (35/478 samples) is comparable to most EPN surveys conducted in tropical and subtropical region, e.g. 6.5% in Nigeria (Akyazi et al., 2012), 6.9% in Ethiopia (Mekete et al., 2005), 7% in Chile (Edgington et al., 2010) and 7% in South Africa (Malan et al., 2006). Since the current survey was the first in this region, it was not targeted toward any habitat type and samples were collected systematically covering all habitat types. Targeted survey of habitats where EPN are more prevalent usually results in higher recovery frequency than random non-targeted survey (Mracek and Becvar, 2000). Therefore, the results of the current survey may be considered as a conservative estimate of EPN occurrence on Viti Levu (Bruck, 2004).

All isolated populations belong to only one species of the genus *Heterorhabditis*. Hominick (2002) had stated that steinernematids are generally recovered more often than *Heterorhabditids* during non-targeted surveys but in the current non-targeted survey of Viti Levu, no steinernematids were encountered. Based on the identity of the ITS sequence, BLAST analysis and phylogenetic study, all native isolates were identified as *Heterorhabditis indica*. Native isolates showed some point variation when compare to each other and type species. These point variations have little relevance at the species level, and are considered to be polymorphic nucleotide variants.

Studies on the morphometric variability are useful to provide valuable information about geographical and ecological requirements for EPN. Morphometric differences can be observed in EPNs strains isolated from different sites and hosts (Campos Herrera *et al*., 2006). The current study shows that total body length of IJ and male are higher than the measurement for type species *H. indica*. Even though the total body length of IJs of native isolates (530-630 μm) is higher in comparison to that in original description (479-573 μm) of *H. indica* by Poinar et al. (1992), it is similar to the body length of *H. indica* isolate reported from Vietnam (490-643 μm) (Phan et al., 2003). Similarly, the average body length of male varied from 748 to 1,137 μm which is higher than reported in original description of *H*. *indica* but similar to that of *H. indica* H.MP16 isolated from Vietnam (929-1,046 μm) (Phan et al., 2003) and *H. hawaiiensis* (864-1,130) which is a junior synonym of *H. indica* (Gardner et al., 1994). When compared to *H. indica* both female and hermaphrodite of native isolates were also much bigger in size. Although their size is much bigger than that of type species, we observed that the average body length for hermaphrodite of native isolates is closer to the average body length (5,000 μm) for *H. hawaiiensis* (=*H. indica*). Similarly, the range for female body length also overlaps with the range reported for *H. hawaiiensis*. Except for D% for hermaphrodite and female, all other morphometric characteristics did not match with those reported for type species *H. indica.* The reason for intraspecific variability in body length could be due to the geographic isolation of these populations (Campos-Herrera et al., 2006; Ivanova et al., 2013; Stock et al., 2000). Stock et al. (2000) and Poinar (1992) showed that the geographical origin and habitat can influence morphometric data of EPN species reared in *G. mellonella*. Additional studies on the morphometric and molecular variability of isolates from different sites and habitats could provide more accurate and valuable insight into this (Stock et al., 2000).

*H. indica* was first isolated from India (Poinar et al., 1992). Since then various studies have demonstrated that *H*. *indica* is cosmopolitan and is widely distributed in tropical and subtropical regions e.g. Brazil (Dolinski et al., 2008), Vietnam (Nguyen and Sturhan, 2006), Benin (Zadji et al., 2013), Cuba, Guadeloupe (Mauleon et al., 2006), Jamaica, Puerto Rico (Roman and Figueroa, 1995), Trinidad, Venezuela (Rosales and Suarez, 1998), India (Razia and Sivaramakrishnan, 2014) and Indonesia (Griffin et al., 2000). Its presence in Oceania has also been documented from Australia and Hawaii (Hominick, 2002). The origin of this species in Fiji Islands is unclear. Fijian isolates may have been inadvertently introduced, as speculated by Akhurst and Bedding (1986) and Hara et al. (1991) for nematodes introduced into Australia and Hawaii. The result of this survey reinforces the reports that *H*. *indica* is more adapted to tropical environment (Hara et al., 1991; Hominick et al., 1996).

The occurrence of *H. indica* in Viti levu showed a highly significant association with climatic zone, annual rainfall, habitat type and soil type. Positive *H. indica* sites occur almost exclusively in the leeward side (rainfall < 180 cm) of the Viti Levu along coastline and riverside. Very few positive sites were found in wet windward side of Island. Windward side of Viti Levu, receives annual rainfall of 305 to 345 cm resulting in very high moisture level which results in the poor aeration and therefore making it unsuitable to EPN, which are obligate aerobes (Mwaniki et al., 2008; Yeh and Alm 1992). The result of this survey supports the finding of other surveys which report dominance of *H. indica* in coastal sandy soils. Around the world, *H. indica* has been mostly recovered from coastal areas and rarely from inland (Phan et al., 2003; Khatri-Chhetri et al., 2010). In this survey, only two agricultural sites were positive, which conforms to the findings of other surveys that reported less prevalence of EPN in agricultural soil (Akhurst and Brooks, 1984; Barker and Barker, 1998; Mekete et al., 2005; Mracek et al., 1999). This could be due to the agricultural practices that result in the disruption of the soil microenvironment and expose the IJs to harmful UV light and desiccation. Agricultural practices are also deleterious to the hosts of EPN through which they recycle themselves (Lawrence et al., 2006). Reduced tillage results in increased prevalence of endemic populations (Brust, 1991) as well as persistence and efficacy of applied EPN (Shapiro et al., 1999). Contrary to this Campos-Herrera et al. (2015) reported no effect of tillage on the occurrence of EPN in Swiss soil, whereas authors like Mracek and Webster (1993) and Mwaniki et al. (2008) have reported the higher occurrence of EPN in agricultural land which they have related to the outbreak of insect population during cropping season. Zepeda-Jazo et al. (2014) also reported higher prevalence of EPN (64.3%) in cultivated habitats and speculated that the soil moisture favored the high recovery of EPNs because the survey was conducted during the rainy season. The recovery frequency of EPN from farmland can be improved by targeting the crop edges which are believed to act as refuge area for the insect pests (Campos-Herrera et al., 2007).

Results of the soil analysis show correlation between soil pH and *H. indica* presence. In the present survey, maximum recovery (12.4%) of the species was from soil with pH ranging from 6.0 to 6.5. The result of this study is in agreement with the studies carried out by Mwaniki et al. (2008) and Rosa et al. (2000) who reported heterorhabditid preferring pH>6. Survey of Guadeloupe islands has reported the isolation of *H*. *indica* from the soil with pH 9.8 (Constant et al., 1998). In current study, the highest value of organic content of positive soil was 11.2%, which is similar to the result reported by Hara et al. (1991) where *Heterorhabditis* presence was reported from the soil with organic content less than 12%. However, statistical analysis of data of the current study does not show any significant association between organic content and *H. indica* presence. Similarly, no correlation was observed between soil moisture level and *H. indica* occurrence.

## Conclusion

This study documents the occurrence of EPNs in Fiji Islands as the first report on EPN. All isolates collected across Viti Levu belong to one species, *Heterorhabditis indica*. *H. indica* was primarily recovered from leeward side of the Viti Levu along the coastline and riversides. The *H. indica* was more prevalent in lighter soil with pH>6. There was significant association between *H. indica* occurrence and average annual rainfall. The study indicates that soil in Viti Levu is rich in *H*. *indica* and therefore, this strain could be potentially important for the biological control of insect pests on the Island.
